# OTME-14. TGF-beta signaling in microtube formation of glioblastoma

**DOI:** 10.1093/noajnl/vdab070.065

**Published:** 2021-07-05

**Authors:** Simon Storevik, Justin Joseph, Capucine Magaut, Luiz Henrique, Thomas Mathivet, Md Abdul Latif, Justine Rudewicz, Joris Guyon, Matteo Gambaretti, Frida Haukås, Amalie Trones, Lars Andreas Rømo Ystaas, Jubayer Hossain, Sandra Ninzima, Wenjing Zhou, Tushar Tomar, Frank Winkler, Frank Kruyt, Andreas Bikfalvi, Rolf Bjerkvig, Thomas Daubon, Hrvoje Miletic

**Affiliations:** 1 University of Bergen, Bergen, Norway; 2 Haukeland University Hospital, Bergen, Norway; 3 Aarhus University, Aarhus, Denmark; 4 University of Bordeaux, Pessac, France; 5 Paris Cardiovascular Research Center, Paris, France; 6 Shandong University, Jinan, China; 7 PamGene International BV, BJ s′-Hertogenbosch, Netherlands; 8 University of Heidelberg, Heidelberg, Germany; 9 University Medical Centre Groningen, Groningen, Netherlands; 10 Luxembourg Institute of Health, Strassen, Luxembourg

## Abstract

Microtubes (MTs) are cytoplasmic extensions of glioma cells serving as important cell communication structures while also promoting invasion and treatment resistance through network formation. MTs are abundant in chemoresistant gliomas, in particular glioblastomas, while they are uncommon in chemosensitive IDH mutated and 1p/19q co-deleted oligodendrogliomas.

By performing a bioinformatics analysis on data from The Cancer Genome Atlas (TCGA) we identified the TGF-b pathway as being distinctly upregulated in glioblastomas compared to oligodendrogliomas, making this a signaling pathway potentially involved in MT formation. Based on patient-derived GBM stem cell line models we demonstrated that stimulation of TGF-b increased MT formation, while inhibition of TGF-b reduced MT formation. MT formation was verified by expression of GAP43 and nestin, which have previously been shown to be important structural proteins of MTs.

Interestingly, we also observed a responder/non-responder relationship between GBM cell lines P3 and GG16/ GG6 regarding MT formation upon TGF-b stimulation. To determine downstream signaling mediators of the TGF-b pathway crucial for MT formation, we subsequently performed RNA sequencing of these cell lines. From the 34 initial candidates common to responders, but absent in non-responders, only 3 genes were left after filtering through TCGA data and *in vivo* RNA sequencing data of a GBM xenograft model derived from P3.

Thrombospondin 1 (TSP1) emerged as the most interesting candidate as we have previously shown that transcription of this gene is activated by TGF-b/SMAD signaling and TSP1 also promotes invasiveness of GBM. TSP1 was upregulated by TGFB1 stimulation in responder cells and promoted MT formation. Transcriptional activation of TSP1 was absent in the non-responder cell line GG6 and could be reversed in the responder cell line P3 by TSP1 shRNAs in vitro and in vivo. Thus, TSP1 was experimentally verified as an important mediator of microtube formation downstream of TGF-b signaling.

